# Immune landscape and biomarker identification in Q fever: a comprehensive diagnostic analysis

**DOI:** 10.1371/journal.pone.0339963

**Published:** 2025-12-30

**Authors:** Zhiyuan Gong, Shixin Liu

**Affiliations:** 1 Tianjin Medical University, Tianjin, China; 2 Department of Clinical Laboratory, Anqiu City People’s Hospital, Weifang, China; Rutgers: Rutgers The State University of New Jersey, UNITED STATES OF AMERICA

## Abstract

**Objectives:**

Q fever (QF), a zoonotic disease caused by *Coxiella burnetii*, exhibits highly heterogeneous clinical manifestations—ranging from asymptomatic infection to acute febrile illness and persistent complications. The immune-related molecular mechanisms underlying its diverse disease stages remain incompletely elucidated. This study aimed to dissect the gene expression dynamics across different QF phases via bioinformatics approaches, thereby uncovering key immune-regulatory molecular mechanisms.

**Methods:**

Gene expression profiles of QF were retrieved from the Gene Expression Omnibus (GEO) database. The CIBERSORT algorithm was employed to quantify the relative proportions of infiltrating immune cells. Temporal gene expression clustering was performed using the mfuzz algorithm to characterize stage-specific immune response patterns. Differentially expressed genes (DEGs) were identified with the limma package, followed by Gene Ontology (GO) and Kyoto Encyclopedia of Genes and Genomes (KEGG) enrichment analyses to explore their biological functions and associated immune pathways. A protein-protein interaction (PPI) network was constructed to screen for core immune-related genes, and Receiver Operating Characteristic (ROC) curve analysis was utilized to evaluate the diagnostic efficacy of these candidate genes for acute Q fever (AQF).

**Results:**

Distinct immune cell infiltration patterns were observed across QF stages: AQF was characterized by elevated proportions of M1 macrophages and activated natural killer (NK) cells, whereas persistent Q fever (PQF) showed a marked increase in M2 macrophages. Temporal clustering analysis revealed that metabolic-related genes were highly expressed in healthy controls, while both AQF and PQF exhibited dynamic upregulation of genes associated with inflammation and immune regulation. Enrichment analyses indicated that the QF-related immune response was closely linked to COVID-19-associated pathways, Th17 cell differentiation, and cytokine-cytokine receptor signaling. Five hub genes (IL6, IL4, IL1B, AKT1, and CD28) were identified in AQF, all of which demonstrated high diagnostic accuracy with favorable Area Under the Curve (AUC) values.

**Conclusion:**

This study delineates the landscape of gene expression and immune status alterations during QF progression. The identified hub genes (IL6, IL4, IL1B, AKT1, and CD28) hold promise as potential diagnostic biomarkers for AQF. Collectively, these findings provide critical insights into the immune-regulatory mechanisms of QF and offer valuable theoretical support for the development of clinical diagnostic tools and therapeutic strategies.

## 1. Introduction

Q fever (Query Fever, QF) is a globally distributed zoonotic disease caused by *Coxiella burnetii* infection, characterized by acute or persistent onset and potentially involving multiple organ systems [1,2]. *C. burnetii* is a Gram-negative obligate intracellular bacterium. Although classified under the order Legionellales, Q fever is commonly grouped with rickettsial diseases and is colloquially referred to as “Q fever rickettsia” [3]. Farm animals (cattle, sheep, goats) and pets serve as primary reservoirs, with ticks as vectors; human infection mainly occurs via inhalation of pathogen-contaminated aerosols [4].

While Q fever incidence is low and sporadic worldwide, its high infectivity and broad host range make it a critical public health concern. Outbreaks may occur under favorable conditions, and immunocompromised individuals face risks of severe complications or persistent infections, burdening healthcare systems [5].

In acute Q fever, the host’s innate immune response is rapidly activated. Phagocytic cells, such as macrophages and dendritic cells, recognize the pathogen and initiate an inflammatory response by releasing cytokines and chemokines, which recruit additional immune cells to the infection site to eliminate the pathogen [6]. However, the bacterium evades immunity (e.g., inhibiting phagosome maturation) to persist intracellularly, driving chronic infection [7]. The acute-to-persistent transition links to immune dysregulation (chronic macrophage activation, T-cell tolerance), with adaptive immune dysfunction (T/B lymphocytes) causing ongoing inflammation and complications like endocarditis [8]. In persistent Q fever, dysfunction of adaptive immune cells, such as T and B lymphocytes, may result in ongoing inflammation and tissue damage, contributing to severe complications such as endocarditis [9]. Against this backdrop, there is an urgent need for systematic approaches to evaluate the role of immune cells in Q fever and to explore immune-related diagnostic biomarkers, which could help elucidate the immunological mechanisms underlying this disease.

Leveraging high-throughput sequencing and bioinformatics, we analyzed peripheral blood transcriptomic data from GEO (Q fever patients) to: [1] compare immune infiltration among healthy controls, acute Q fever (AQF), and persistent Q fever (PQF) via CIBERSORT; [2] identify immunity-associated AQF differentially expressed genes (IAQDEGs) and explore their functional enrichment; [3] screen candidate diagnostic biomarkers using multiple algorithms.”

This study aims to lay a bioinformatics foundation for understanding Q fever pathogenesis, identify novel diagnostic biomarkers, and provide insights for its diagnosis and immunotherapy.

## 2. Materials & methods

### 2.1. Data sources and processing

We downloaded the gene expression profile of Q fever (GSE112086) from the Gene Expression Omnibus (GEO) database (https://www.ncbi.nlm.nih.gov/geo/), which was generated based on the GPL6480 platform. The samples included in this study are detailed in Table 1. All samples used in this study are de-identified public gene expression data and do not contain private information that can identify individual subjects. According to the relevant regulations of the Ethics Committee of Anqiu City People’s Hospital (the affiliated institution), secondary analyses based on public de-identified databases do not require separate ethical approval. Additionally, this study does not involve any additional experiments on animals, humans, or other experimental subjects.

**Table 1:** Information and grouping of the samples.

Group	Accession	Title	Source name	Subject status	Tissue
Control	GSM3057232	Control_1	Healthy control	Healthy control	Whole blood
Control	GSM3057233	Control_2	Healthy control	Healthy control	Whole blood
Control	GSM3057234	Control_3	Healthy control	Healthy control	Whole blood
Control	GSM3057235	Control_4	Healthy control	Healthy control	Whole blood
Control	GSM3057236	Control_5	Healthy control	Healthy control	Whole blood
AQF	GSM3057216	AQF_1	Acute Q Fever patient	Acute Q fever	Whole blood
AQF	GSM3057217	AQF_2	Acute Q Fever patient	Acute Q fever	Whole blood
AQF	GSM3057218	AQF_3	Acute Q Fever patient	Acute Q fever	Whole blood
AQF	GSM3057219	AQF_4	Acute Q Fever patient	Acute Q fever	Whole blood
AQF	GSM3057220	AQF_5	Acute Q Fever patient	Acute Q fever	Whole blood
AQF	GSM3057221	AQF_6	Acute Q Fever patient	Acute Q fever	Whole blood
AQF	GSM3057222	AQF_7	Acute Q Fever patient	Acute Q fever	Whole blood
AQF	GSM3057223	AQF_8	Acute Q Fever patient	Acute Q fever	Whole blood
PQF	GSM3057224	PQF_1	Persistent Q fever patient	Persistent Q fever	Whole blood
PQF	GSM3057225	PQF_2	Persistent Q fever patient	Persistent Q fever	Whole blood
PQF	GSM3057226	PQF_3	Persistent Q fever patient	Persistent Q fever	Whole blood
PQF	GSM3057227	PQF_4	Persistent Q fever patient	Persistent Q fever	Whole blood
PQF	GSM3057228	PQF_5	Persistent Q fever patient	Persistent Q fever	Whole blood
PQF	GSM3057229	PQF_6	Persistent Q fever patient	Persistent Q fever	Whole blood
PQF	GSM3057230	PQF_7	Persistent Q fever patient	Persistent Q fever	Whole blood
PQF	GSM3057231	PQF_8	Persistent Q fever patient	Persistent Q fever	Whole blood

The gene expression matrix was processed using R software (version 4.2.2) for probe annotation, duplication removal, and log-transformation normalization to standardize the data.

### 2.2. Estimation of immune cell infiltration

CIBERSORT is a computational method widely applied for immune infiltration analysis based on gene expression data [10]. This algorithm uses a deconvolution approach with support vector regression to quantify the relative proportions of 22 different immune cell types within a mixed cell population, relying on the predefined LM22 signature matrix (a reference gene expression profile of 22 human immune cell subsets, Supplementary Material 1). For the present analysis, CIBERSORT was run with 1000 permutations to assess the statistical significance of deconvolution results, and only samples with a deconvolution P-value < 0.05 were retained for subsequent analysis to ensure the reliability of immune cell proportion estimates.

The CIBERSORT algorithm was applied to estimate the proportions of different immune cells in the samples and analyze the immune cell composition of healthy individuals, acute Q fever patients, and persistent Q fever patients. Box plots were used to visualize the immune cell distributions between the groups. Differences in immune cell proportions were assessed using the Wilcoxon rank-sum test, with results considered statistically significant at *P* < 0.05.

### 2.3. Identification of differentially expressed genes (DEGs)

DEGs were analyzed using the R software package limma [11]. The limma workflow involves fitting linear models with lmFit, moderating t-statistics via empirical Bayes shrinkage with eBayes, and subsequently calculating adjusted *P*-values (FDR) using the Benjamini-Hochberg (BH) method by default in the topTable function (adjust.method = “BH”). The screening criteria for differential genes were: |log_2_FC| > 1.0 and *P* < 0.05. The term “immune” was queried in the GeneCards database [12] (https://www.genecards.org/) to identify relevant genes. Based on the search results, genes with a GeneCards score >10 were classified as immune-related genes (IRGs) (Supplementary Material 2). The immune-related and acute Q fever-related differentially expressed genes (IAQDEGs) were obtained by intersecting the DEGs with the IRGs.

### 2.4. Functional and pathway enrichment analysis

Gene ontology (GO) functional annotation and Kyoto encyclopedia of genes and genomes (KEGG) pathway analysis were performed using the DAVID database [13] (https://david.ncifcrf.gov/home/jsp). GO function annotation include three parts: molecular function (MF), biological process (BP), and cell component (CC). The threshold set for GO and KEGG enrichment analyses was *P* < 0.05.

### 2.5. Construction of the protein-protein interaction (PPI) network and screening of hub genes

IAQDEGs were imported into the STRING database [14] (https://string-db.org/), and a confidence level> 0.4 was selected for PPI network analysis to build a visual PPI network. PPI network data files were imported into Cytoscape (V3.9.1) software and the top 10 hub genes were calculated using the 10 algorithms (Betweenness, Closeness, Degree, EPC, MCC, MNC, Radiality and Stress) in its plug-in cytoHubba [15].

### 2.6. Assessment of candidate biomarkers for diagnostic accuracy

The effectiveness of the candidate biomarkers for the diagnosis of acute Q fever was validated using ROC logistic regression implemented via the pROC package [16] in R software. The area under the ROC curve (AUC) was used as the evaluation criterion.

### 2.7. Mfuzz algorithm

Fuzzy c-means clustering (mfuzz algorithm) was used to analyze gene expression patterns across discrete disease states (Control, AQF, PQF), implemented via the R package ClusterGVis (v0.1.2) in R software (v4.3.2). A fixed random seed (seed = 5201314) was set to ensure result reproducibility. The input data was the preprocessed gene expression matrix (rows = genes, columns = samples ordered as Control, AQF, PQF). The optimal cluster number was determined as 8 based on three criteria: ①elbow method (inflection point of within-cluster sum of squares (WSS) curve at k = 8); ②maximum Silhouette coefficient (0.72, indicating optimal clustering compactness and separation); ③alignment with Q fever’s gene expression characteristics to distinguish functional modules (metabolism, inflammation, immune regulation). Clustering was performed using the `clusterData` function with `cluster.method = “mfuzz”` and `cluster.num = 8`, generating a clustering object containing gene cluster assignments and expression trend data for subsequent visualization and functional enrichment analysis.

### 2.8. Relationship between diagnostic biomarkers and infiltrating immune cells

The Pearson correlation coefficient, a statistical measure that quantifies the linear relationship between two random variables, was used to analyze the association between candidate diagnostic biomarkers and infiltrating immune cells in AQF. Correlations were considered significant and meaningful if the absolute value of the correlation coefficient (|R|) was ＞ 0.5 and the *P*-value was < 0.05.

### 2.9. Data visualization

The data visualization in this study was performed using San**gerBox** [17] (http://vip.sangerbox.com/home.html), Weishengxin [18] (http://www.bioinformatics.com.cn/), and R software.

## 3. Results

### 3.1. Analysis of immune cell infiltration in Q fever versus healthy samples

**Fig 1:**
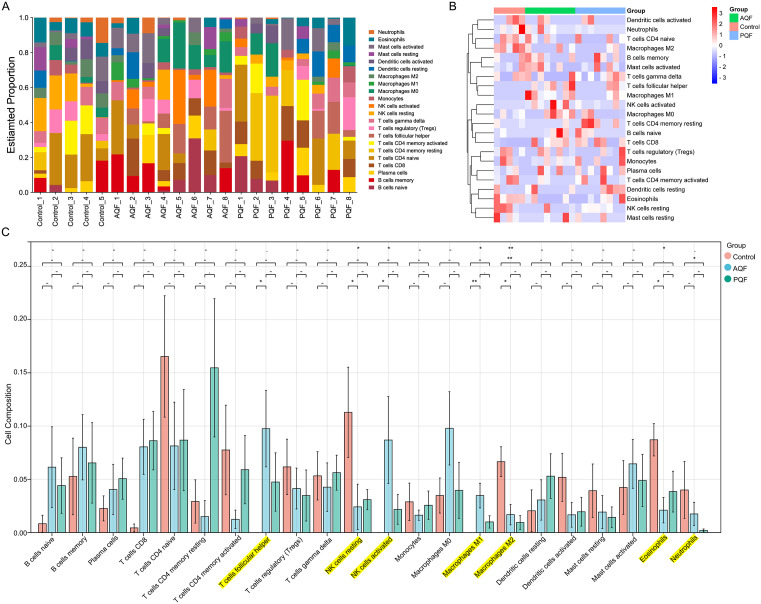
Immune cell infiltration in Q fever and healthy samples. **(A)** Stacked bar plot illustrating the proportional composition of 22 immune cell types across 21 samples (Control: 5 cases, AQF: 8 cases, PQF: 8 cases). Samples were grouped as Control (healthy individuals), AQF (acute Q fever patients), and PQF (persistent Q fever patients). **(B)** Heatmap depicting the distribution of immune cell proportions in the three groups, with rows representing immune cell types and columns representing individual samples. **(C)** Bar plot highlighting the differences in immune cell proportions among the three groups. Analysis was performed using the CIBERSORT algorithm to estimate immune cell infiltration, and statistical significance was determined by the Wilcoxon rank-sum test. Symbols: * P < 0.05, ** P < 0.01 (statistically significant differences); – P > 0.05 (no significant difference).

Immune cell infiltration analysis is widely used to study the pathogenesis of diseases. In this study, we utilized the CIBERSORT algorithm to calculate the proportions of different immune cells in the samples, analyzing the immune cell composition of healthy individuals, acute Q fever patients, and persistent Q fever patients. Fig 1A depicts the proportional composition of immune cell types across the three groups. The control group demonstrates a balanced immune state, with neutrophils and monocytes being the predominant immune cell types. In the AQF group, there is an observed increase in the proportions of M1 macrophages and activated NK cells, suggesting a trend toward acute inflammatory responses. The PQF group shows a shift in immune composition, with a higher proportion of M2 macrophages and a lower proportion of neutrophils, which may indicate immune regulation or a chronic inflammatory state. Fig 1B, presented as a heatmap, further highlights these trends, with the Control group maintaining immune homeostasis, the AQF group dominated by inflammation-associated cells, and the PQF group showing a predominance of immune-regulatory cells. Further statistical results show that eosinophils are significantly reduced in both the AQF and PQF groups, suggesting that pathological conditions may suppress their recruitment or activity. In the AQF group, T follicular helper cells, activated NK cells, and M1 macrophages are significantly increased, reflecting the immune response characteristic of acute inflammation. In contrast, the PQF group shows a significant increase in M2 macrophages and a significant decrease in neutrophils, indicating features of chronic inflammation and immune suppression (Fig 1C).

### 3.2. Gene expression characteristics of healthy people, acute Q fever, and persistent Q fever patients

**Fig 2:**
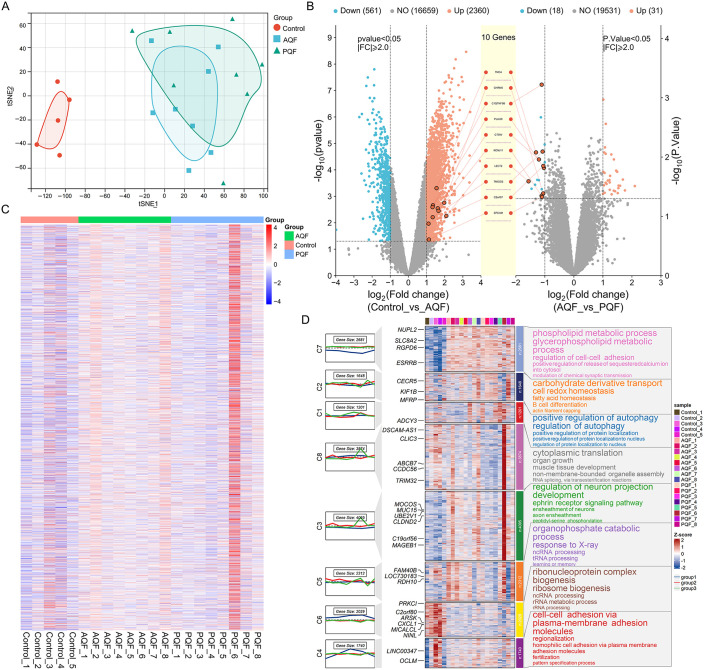
Gene expression characteristics of the three sample groups. **(A)** t-SNE plot of gene expression profiles across 21 samples (Control: 5 cases, AQF: 8 cases, PQF: 8 cases) after dimensionality reduction. Each dot represents an individual sample, with colors distinguishing the three groups (Control: healthy individuals, AQF: acute Q fever patients, PQF: persistent Q fever patients). **(B)** Dual volcano plots showing differential gene expression between Control vs AQF (left) and AQF vs PQF (right). Genes were screened using the limma package with the criteria |log2FC| > 1.0 and P < 0.05; red dots indicate upregulated genes, blue dots indicate downregulated genes, and gray dots indicate non-differential genes. The yellow region highlights 10 core genes with significant changes in both comparisons. **(C)** Heatmap of differentially expressed genes (DEGs) across the three groups, with rows representing DEGs and columns representing samples, and color intensity indicating normalized gene expression levels. **(D)** Gene expression clustering and functional enrichment analysis based on the mfuzz algorithm. Note: The algorithm was applied to cluster gene expression patterns across the three cross-sectional groups (not true time-series data), with each cluster representing genes with consistent expression trends; functional enrichment was performed using KEGG pathway analysis (P < 0.05).

Using the t-SNE (t-distributed stochastic neighbor embedding) dimensionality reduction method, clustering analysis was performed on the samples from the Control, AQF, and PQF groups (Fig 2A). The Control group forms a distinct and tight cluster in the t-SNE space, indicating highly similar gene expression characteristics within this group. In contrast, the AQF group shows a more dispersed clustering pattern compared to the Control group, reflecting a certain degree of heterogeneity in gene expression among acute Q fever patients. The PQF group exhibits an even broader distribution, with partial overlap with the AQF group, suggesting that the gene expression characteristics of persistent Q fever patients share some similarities with AQF while also displaying unique changes. These results indicate that the Control group and the disease groups (AQF and PQF) have distinct gene expression patterns, while AQF and PQF exhibit a degree of transition and connection in their gene expression profiles.

The dual volcano plots further illustrate the differences in gene expression between the groups (Fig 2B). Compared to the Control group, the AQF group shows a significantly higher number of upregulated genes than downregulated genes, suggesting that acute Q fever may involve widespread activation of gene expression. In contrast, the number of upregulated and downregulated genes in PQF compared to AQF is significantly reduced (a total of 49 genes), indicating that the differences in gene expression between persistent and acute Q fever are relatively small but still include key gene expression changes. The yellow region in the middle highlights 10 core genes that show significant changes in both Control vs. AQF and AQF vs. PQF comparisons, which may serve as important biomarkers for dynamically monitoring the progression of Q fever stages. Subsequent heatmaps illustrate the differences in gene expression patterns among the Control, AQF, and PQF groups. The Control group shows distinct separation from the disease groups, while AQF and PQF share similarities in certain gene expression characteristics (Fig 2C).

Finally, the mfuzz algorithm was utilized to perform clustering analysis based on gene expression patterns across the three cross-sectional groups (Control, AQF, and PQF), aiming to identify gene clusters with consistent expression characteristics that distinguish the three distinct states (healthy, acute Q fever, and persistent Q fever) (Fig 2D). In the Control group, genes related to metabolism and biosynthesis (e.g., Cluster 1) exhibited high expression levels. These genes were significantly enriched in pathways such as phospholipid metabolism and glycoprotein biosynthesis, but were markedly downregulated in both the AQF and PQF groups, suggesting that these metabolic pathways may be suppressed under Q fever pathological conditions. This expression pattern reflects the normal metabolic activity characteristic of healthy individuals. The AQF group was characterized by a significant upregulation of genes associated with inflammation and immune activation (e.g., Cluster 2). These genes, closely related to cell-cell adhesion and inflammatory responses, showed high expression levels, reflecting the inflammatory nature of acute Q fever and the heightened immune activation during this stage. The PQF group displayed a distinct upregulation of genes related to immune regulation and tissue repair (e.g., Cluster 4). These genes were enriched in pathways such as autophagy regulation and neuronal projection development, suggesting that autophagy and tissue repair mechanisms may play crucial roles in the pathology of persistent Q fever. This expression pattern highlights the chronic immune regulation and pathological state associated with PQF.

### 3.3. Identification of IAQDEGs

**Fig 3:**
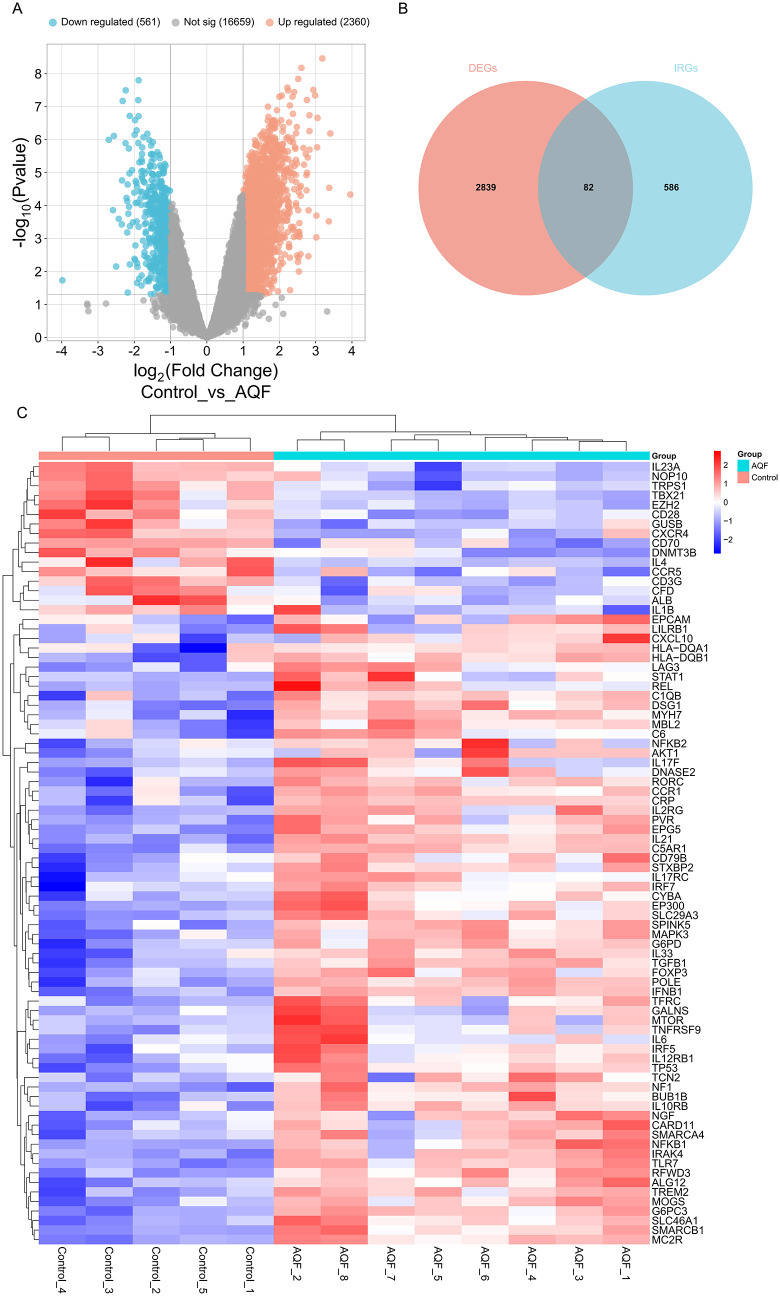
Identification of IAQDEGs in AQF. **(A)** Volcano plot of DEGs between AQF (8 cases) and Control (5 cases) samples. DEGs were identified using the limma package with the criteria |log2FC| > 1.0 and P < 0.05; red dots represent upregulated genes, blue dots represent downregulated genes, and gray dots represent non-differential genes. **(B)** Venn diagram showing the intersection of AQF-related DEGs (2,921 genes) and immune-related genes (IRGs, 668 genes retrieved from the GeneCards database with a score > 10). The overlapping region represents immune-related acute Q fever differentially expressed genes (IAQDEGs, 82 genes). **(C)** Heatmap of 82 IAQDEGs, with rows representing genes and columns representing samples (Control: 5 cases, AQF: 8 cases), and color intensity indicating normalized gene expression levels (red: high expression, blue: low expression).

A total of 2,921 DEGs were identified between AQF samples and normal samples, including 2,360 upregulated and 561 downregulated genes in AQF samples (Fig 3A). Meanwhile, 668 immune-related genes were retrieved from the GeneCards database. By intersecting the 2,921 DEGs with the 668 immune-related genes, 82 IAQDEGs were obtained (Fig 3B), comprising 66 upregulated genes and 16 downregulated genes (Fig 3C).

### 3.4. Functional enrichment and pathway analysis of IAQDEGs

**Fig 4:**
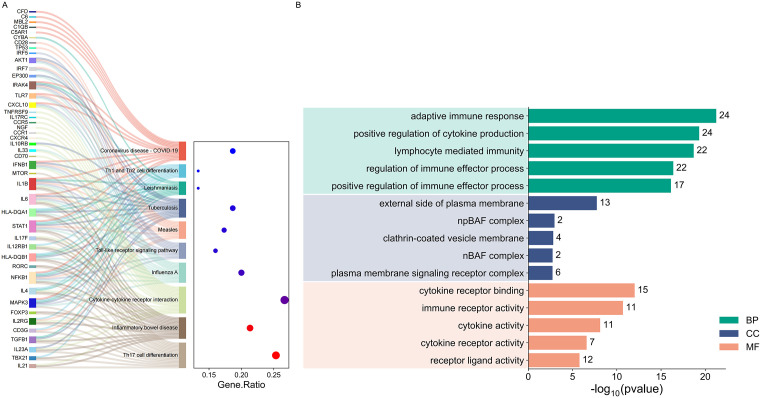
GO and KEGG pathway analysis of IAQDEGs. **(A)** KEGG pathway analysis of 82 IAQDEGs. The x-axis represents the enrichment score (–log10 P-value), and the y-axis represents enriched pathways. Analysis was performed using the DAVID database with a statistical threshold of P < 0.05. **(B)** GO functional annotation of 82 IAQDEGs, including three dimensions: Biological Process (BP), Cellular Component (CC), and Molecular Function (MF). The x-axis represents the number of genes enriched in each term, and the y-axis represents specific GO terms. Analysis was performed using the DAVID database with a statistical threshold of P < 0.05.

GO and KEGG enrichment analyses are important tools for extracting biological information from genomic data. By analyzing gene functions and pathway contexts, they reveal the roles of genes in specific biological processes, providing crucial support for disease research, mechanism exploration, and target discovery. To further explore the immune-related biological processes and functional changes involved in the development of Q fever, we performed KEGG and GO enrichment analyses on IAQDEGs. KEGG analysis showed that the molecular mechanisms of acute Q fever (AQF) are closely related to various immune-related pathways, including the COVID-19-related pathway and the Th17 cell differentiation pathway, highlighting the critical role of Th17 cells in the inflammatory response of acute Q fever; and the cytokine-cytokine receptor interaction pathway, emphasizing the central role of cytokine signaling in immune responses (Fig 4A). GO functional annotation further described the roles of IAQDEGs in immune processes from three dimensions: Biological Process (BP), Cellular Component (CC), and Molecular Function (MF). The results revealed that these genes play multi-level roles in adaptive immunity, cytokine regulation, signal transduction, and antigen presentation, with particular enrichment in the extracellular matrix and MHC protein complexes, suggesting the important role of dynamic immune cell regulation in the pathological process of Q fever (Fig 4B). These findings provide important insights for further studying the molecular mechanisms of Q fever and lay the foundation for the discovery of potential therapeutic targets.

### 3.5. Construct PPI network and screen hub genes

**Fig 5:**
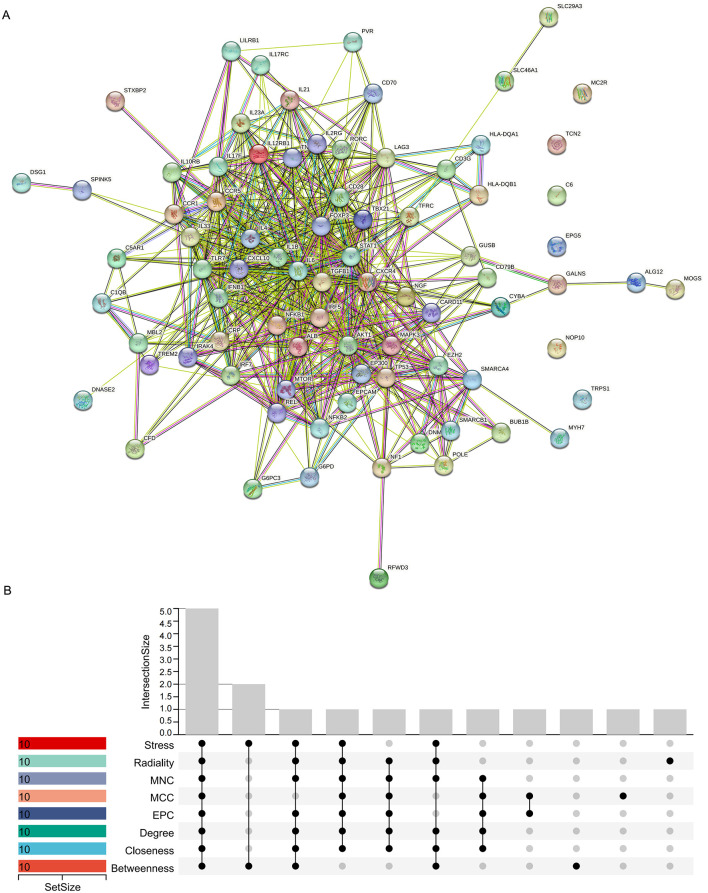
Screening of hub genes. **(A)** Protein-protein interaction (PPI) network of 82 IAQDEGs constructed using the STRING database with a confidence level > 0.4. Nodes represent proteins (genes), and edges represent interaction relationships between proteins. **(B)** Identification of key hub genes using the UpSet algorithm. The plot shows the intersection of top 10 hub genes identified by 10 algorithms (Betweenness, Closeness, Degree, EPC, MCC, MNC, Radiality, Stress) in the cytoHubba plugin of Cytoscape software. The five overlapping genes (IL4, IL6, IL1B, AKT1, CD28) were defined as key hub genes.

IAQDEGs were input into the STRING database to construct a PPI network (Fig 5A). PPI network data were imported into Cytoscape software, and the top hub genes were identified using the built-in cytoHubba plugin, which applies 10 different algorithms to individually screen for 10 hub genes (Table 2). Subsequently, the “UpSet” package was used to intersect the results, leading to the identification of five key hub genes: IL4, IL6, IL1B, AKT1, and CD28 (Fig 5B).

**Table 2:** Ranking of hub genes identified by different algorithms.

	Betweenness	Closeness	Degree	EPC	MCC	MNC	Radiality	Stress
1	IL1B	IL1B	IL1B	IL1B	IL1B	IL1B	IL1B	IL1B
2	IL6	IL6	IL6	IL6	IL6	IL6	IL6	IL6
3	GUSB	CXCL10	CXCL10	NFKB1	NFKB1	CXCL10	AKT1	GUSB
4	AKT1	AKT1	AKT1	CXCL10	CXCL10	AKT1	STAT1	AKT1
5	STAT1	STAT1	STAT1	AKT1	AKT1	STAT1	IL4	STAT1
6	IL4	IL4	IL4	STAT1	IL4	IL4	FOXP3	IL4
7	GALNS	FOXP3	FOXP3	IL4	FOXP3	FOXP3	TGFB1	GALNS
8	CD28	TGFB1	TGFB1	FOXP3	TGFB1	TGFB1	CD28	TGFB1
9	TFRC	CD28	CD28	TGFB1	CD28	CD28	ALB	CD28
10	TP53	TP53	TP53	CD28	IFNB1	TP53	TP53	TP53

### 3.6. Evaluation of the diagnostic accuracy of candidate biomarkers in AQF

**Fig 6:**
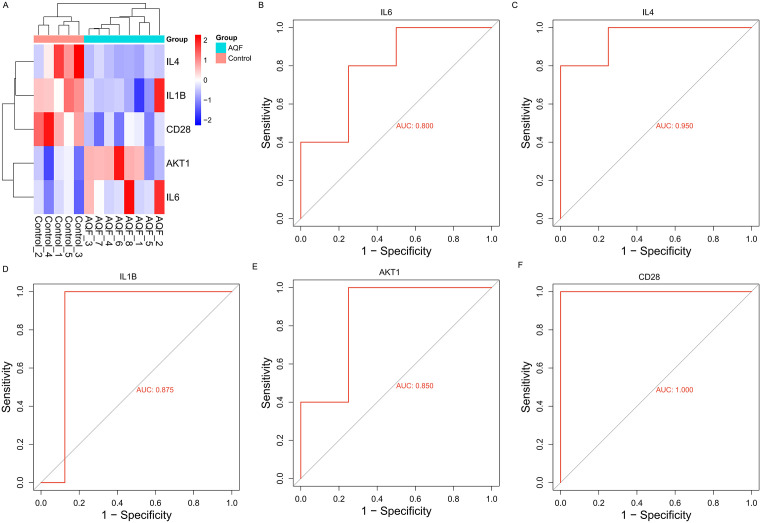
Validation of the 5 Hub genes in AQF diagnosis with ROC curve analysis. **(A)** Heatmap of the five key hub genes (IL4, IL6, IL1B, AKT1, CD28) in 21 samples (Control: 5 cases, AQF: 8 cases, PQF: 8 cases). Color intensity indicates normalized gene expression levels (red: high expression, blue: low expression). **(B-F)** ROC curves of the five key hub genes for diagnosing AQF (8 cases) vs Control (5 cases). Analysis was performed using the pROC package in R software, with the area under the curve (AUC) as the evaluation criterion for diagnostic accuracy. AUC values: IL6 = 0.800, IL4 = 0.950, IL1B = 0.875, AKT1 = 0.850, CD28 = 1.000.

Among the five key hub genes, IL6 and AKT1 were upregulated in the AQF group, while IL4, IL1B, and CD28 were downregulated in the AQF group (Fig 6A). To assess the diagnostic effectiveness of these five hub genes in AQF, we performed ROC logistic regression analysis using the pROC package based on gene expression data. The AUC values for IL6, IL4, IL1B, AKT1, and CD28 were 0.800, 0.950, 0.875, 0.850, and 1.000, respectively (Fig 6B-6F), indicating that these five hub genes exhibit good sensitivity and specificity for the diagnosis of AQF.

### 3.7. Association between biomarkers and immune cell infiltration

**Fig 7:**
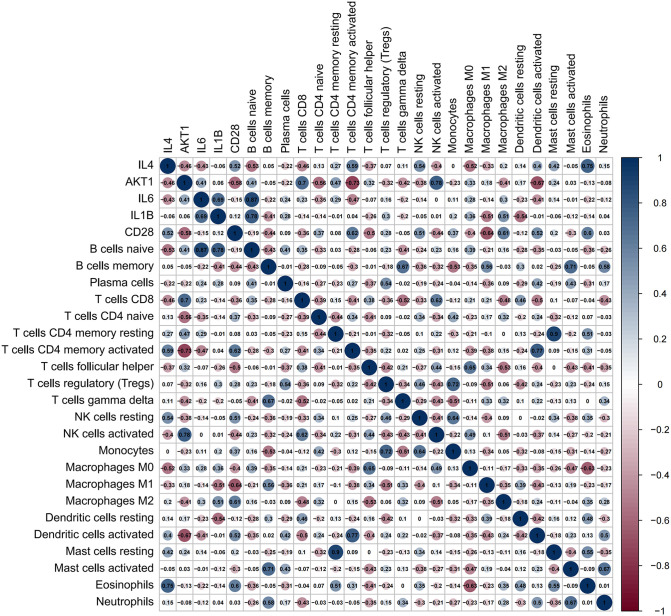
Heatmap of the correlation between candidate diagnostic biomarkers and immune infiltrating cells. Heatmap showing the Pearson correlation coefficients between five candidate diagnostic biomarkers (IL6, IL4, IL1B, AKT1, CD28) and 22 immune infiltrating cell types in AQF patients (8 cases). The color gradient represents the correlation coefficient (red: positive correlation, blue: negative correlation). Correlation analysis was performed using the Pearson correlation coefficient, with statistical significance defined as |R| > 0.5 and P < 0.05.

**Fig 8:**
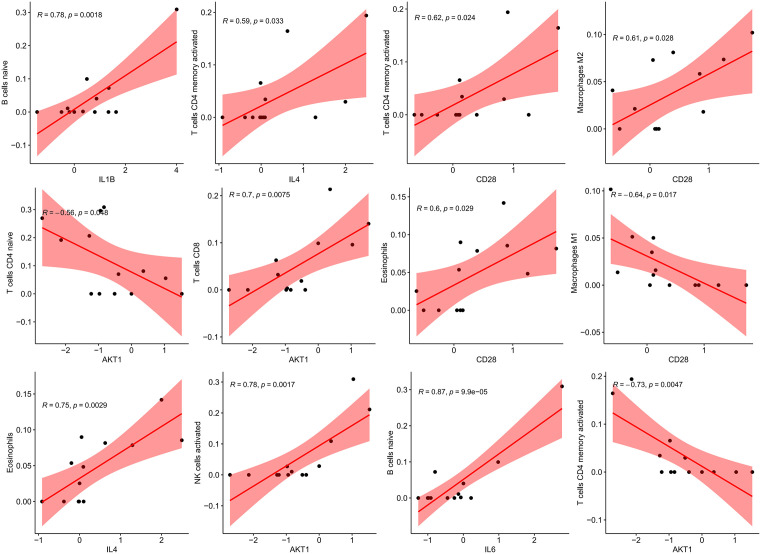
Scatter plot of the correlation between candidate diagnostic biomarkers and immune infiltrating cells. Scatter plots visualizing statistically significant correlations (|R| > 0.5 and P < 0.05) between five candidate diagnostic biomarkers (IL6, IL4, IL1B, AKT1, CD28) and key immune infiltrating cell types in AQF patients (8 cases). Each dot represents an individual AQF sample. The x-axis represents gene expression levels (normalized), and the y-axis represents immune cell proportions (estimated by CIBERSORT). The black line indicates the linear regression trend, with R (Pearson correlation coefficient) and P-values labeled in each plot.

Finally, we analyzed the associations between five candidate diagnostic biomarkers (IL6, IL4, IL1B, AKT1, and CD28) and 22 different immune infiltrating cells (Fig 7). Scatter plots were then constructed to visualize the statistically significant associations (Fig 8). Specifically, IL1B was positively correlated with naive B cells (R = 0.78, p = 0.0018) and activated CD4 + memory T cells (R = 0.59, p = 0.033), indicating that as IL1B expression increases, the numbers of these immune cell populations also increase. IL4 showed a positive correlation with activated CD4 + memory T cells (R = 0.62, p = 0.024) and M2 macrophages (R = 0.61, p = 0.028), suggesting that IL4 likely plays a significant role in promoting these immune cells. AKT1 was negatively correlated with naive CD4 + T cells (R = −0.56, p = 0.08), indicating that higher AKT1 expression may be associated with a decrease in T cell populations, although this result was marginally significant. CD28 exhibited significant correlations with multiple immune cell types, notably a positive correlation with activated CD4 + memory T cells (R = 0.62, p = 0.029) and a negative correlation with M1 macrophages (R = −0.64, p = 0.017), suggesting the potential role of CD28 in immune regulation. IL6 was strongly positively correlated with eosinophils (R = 0.75, p = 0.0029) and activated NK cells (R = 0.78, p = 0.0017), indicating a close association between IL6 expression and the number of these immune cells.

## 4. Discussion

Q Fever is a complex zoonotic disease caused by infection with C. burnetii [19]. It is potentially driven by persistent infection and immune dysregulation, which result from the interplay of innate and adaptive immune responses. This dysregulation may lead to long-term tissue damage and inflammation, contributing to severe complications such as endocarditis [20–23]. Numerous studies have highlighted the critical role of immune cell infiltration and interaction in the pathogenesis of Q fever and the establishment of persistent infection [24,25]. Therefore, modulating immune responses is essential for the effective treatment of Q fever. However, the complex and highly heterogeneous disease course of Q fever has posed challenges for the identification of effective diagnostic biomarkers and the development of systematic treatment strategies. High-throughput sequencing technology provides new opportunities to investigate the mechanisms underlying the onset and progression of Q fever and to identify novel diagnostic biomarkers. In this study, we combined high-throughput sequencing with bioinformatics analyses to reveal, for the first time, the characteristics of immune cell infiltration and gene expression patterns in acute and persistent Q fever. Moreover, we identified key regulatory factors potentially associated with Q fever progression. These findings offer significant insights into the immunopathological mechanisms of Q fever and provide a robust foundation for the development of effective diagnostic and therapeutic approaches.

This study, based on the CIBERSORT algorithm, analyzed the immune cell composition of AQF and PQF patients, revealing the dynamic changes in host immune responses during C. burnetii infection and further elucidating the possible mechanisms by which the pathogen regulates host immune mechanisms to achieve persistent infection. In the AQF group, the proportion of M1 macrophages and activated NK cells significantly increased, indicating that acute inflammatory responses dominated during the early stage of the disease. M1 macrophages exert bactericidal effects by secreting pro-inflammatory factors such as tumor necrosis factor-alpha (TNF-α), interleukin-1β (IL-1β), and nitric oxide (NO), a hallmark of acute infection [26–28]. Furthermore, studies have suggested that C. burnetii, as a Gram-negative bacterium, may enhance the clearance of pathogens by activating Toll-like receptors (TLRs) and the NF-κB signaling pathway to polarize host macrophages toward the M1 phenotype [29].

Notably, the mTOR signaling pathway may play an important regulatory role in this transformation process—as a core hub for immune cell metabolism and activation, it may integrate the TLR/NF-κB signals activated by Coxiella burnetii and nutrient-sensing signals, thereby promoting the glycolytic metabolic reprogramming of M1 macrophages and providing energy and metabolic basis for the secretion of pro-inflammatory factors [30–35]. The activation of NK cells further amplified this pro-inflammatory response, with these cells inhibiting pathogen spread by secreting interferon-gamma (IFN-γ) and directly lysing infected host cells [36]. Despite the strong immune activation observed in the acute phase, C. burnetii can suppress inflammation and limit immune clearance by regulating autophagy and immune evasion mechanisms within host cells [29]. As a classic negative regulator of autophagy [37–40], it is hypothesized that the pathogen might interfere with the autophagic process by targeting and regulating the mTOR signaling pathway, thereby achieving immune evasion.

In contrast to AQF, the immune response in the PQF group exhibited characteristics of immune regulation and chronic inflammation. The proportion of M2 macrophages significantly increased, while the number of neutrophils markedly decreased. M2 macrophages typically suppress pro-inflammatory responses and promote tissue repair by secreting anti-inflammatory factors such as interleukin-10 (IL-10) and transforming growth factor-beta (TGF-β) [41,42]. Previous studies have shown that C. burnetii can induce atypical M2 activation by upregulating genes associated with M2 polarization, such as TGF-β1, IL-1 receptor antagonists, and mannose receptors, thereby suppressing the host’s anti-infective immunity [43]. Notably, this persistent infection is not only closely related to the polarization characteristics of macrophages but may also be influenced by the functional status of monocytes. Evidence suggests that C. burnetii has evolved complex mechanisms to disrupt phagocytosis, such as interfering with integrin interactions in host cells [24]. Additionally, the secretion of IL-10 by monocytes has been identified as a significant driver of chronic Q fever, with studies in mouse models further confirming the critical role of IL-10 in the persistence of C. burnetii [24]. Moreover, it has been demonstrated that the uptake of apoptotic cells by circulating monocytes may redirect these cells toward a non-protective M2-polarized state, thereby enhancing pathogen replication [24]. Under conditions of interferon-gamma, this M2 polarization state is suppressed, and monocytes are more likely to adopt an M1 program, enhancing pathogen clearance. This phenomenon is consistent with the immune features observed in acute Q fever patients [24]. Additionally, this study found that the proportion of eosinophils was significantly reduced in both AQF and PQF, suggesting that their role in the pathological process of Q fever may be limited. The immune dynamics between AQF and PQF patients reflect the complex immunoregulatory mechanisms in C. burnetii infection. The acute phase is primarily dominated by pro-inflammatory immune cells, such as M1 macrophages and activated NK cells, demonstrating strong inflammatory responses. In contrast, the persistent phase is characterized by the predominance of regulatory immune cells, such as M2 macrophages, highlighting the disrupted balance of immune suppression in chronic infections. These findings not only deepen our understanding of the immunopathological mechanisms of Q fever but also provide a theoretical basis for future immunotherapeutic strategies targeting chronic Q fever.

To identify more effective diagnostic biomarkers, we screened 2,921 DEGs between acute Q fever (AQF) and normal samples, including 82 IAQDEGs. GO and KEGG pathway analyses further highlighted the critical roles of these IAQDEGs in immune-related biological processes and functional changes. KEGG analysis revealed that the molecular mechanisms of AQF were closely associated with multiple immune-related pathways, including the COVID-19 pathway, suggesting that Q fever might share similar immune mechanisms with COVID-19, such as intense inflammatory responses and excessive immune activation; the Th17 cell differentiation pathway, emphasizing the crucial role of Th17 cells in AQF inflammatory responses; and the cytokine-cytokine receptor interaction pathway, underscoring the central role of cytokine signaling in immune responses. GO functional annotation provided a comprehensive description of IAQDEGs in immune processes across three dimensions: Biological Process (BP), Cellular Component (CC), and Molecular Function (MF). In the BP dimension, the genes were significantly enriched in adaptive immune responses, cytokine regulation, lymphocyte-mediated immunity, and the regulation of immune effector processes, indicating their essential roles in modulating immune system functions and responses. In the CC dimension, the genes were prominently enriched in cellular components such as the external side of the plasma membrane, the nucleoplasmic complex, and coated vesicle membranes, suggesting their involvement in cellular signal transduction, antigen presentation, and inflammatory responses. In the MF dimension, the genes were enriched in functions such as cytokine receptor binding, immune receptor activity, and ligand activity, further emphasizing the importance of cytokine signaling in the pathological process of Q fever.

Subsequently, we utilized 10 algorithms from the cytoHubba plugin in Cytoscape to identify five hub genes: IL4, IL6, IL1B, AKT1, and CD28. These hub genes were validated using ROC logistic regression analysis, demonstrating their robust diagnostic performance and further strengthening the reliability of our study results. We hypothesize that these five hub genes play pivotal regulatory roles in the pathogenesis of AQF. These findings provide valuable insights into the molecular mechanisms of Q fever and lay a solid foundation for identifying potential therapeutic targets. Collectively, our results not only enhance the understanding of Q fever pathogenesis but also provide essential leads for future experimental validation.

Although this study provides an exploratory perspective on Q fever research by supplementing data on the molecular characteristics of acute Q fever (AQF) and persistent Q fever (PQF), it still has several limitations. Firstly, the GSE112086 dataset is a cross-sectional design comprising three independent populations (healthy controls, AQF, and PQF patients) with no inherent time-point or individual follow-up data. The mfuzz algorithm, traditionally designed for time-series data, was applied here to cluster gene expression patterns across the three discrete states to identify distinguishing trends; however, results only reflect inter-group differences, not true temporal dynamics or disease progression, and longitudinal data scarcity limits causal inference. Secondly, Q fever’s sporadic nature hinders sample collection, resulting in a limited sample size that may compromise the generalizability and reliability of findings. Thirdly, CIBERSORT estimates immune cell proportions using bulk gene expression matrices containing candidate cytokine genes (e.g., IL4, IL6, IL1B), introducing a potential autocorrelation risk and circular reasoning bias in the correlation analysis between biomarkers and immune infiltrates, which requires cautious interpretation. Fourthly, the study lacks comprehensive clinical details (e.g., disease course, comorbidities, treatment history), and the five identified core genes (IL4, IL6, IL1B, AKT1, and CD28) are common universal immune hub genes and candidate biomarkers across multiple diseases. Current data only confirm their involvement in Q fever-related immune responses but not their specificity for Q fever. Additionally, this research relies solely on bioinformatics analyses, with no in vitro or in vivo validation of the biomarkers and mechanisms. Future studies should expand the sample size, integrate detailed clinical data, perform experimental validation, conduct comparative analyses with other infectious diseases to clarify the differential diagnostic value of these candidates, and adopt longitudinal designs to facilitate the clinical translation of Q fever-related diagnostic and therapeutic strategies.

In summary, this study is the first to employ bioinformatics methods to reveal the characteristics of immune cell infiltration and gene expression patterns associated with acute Q fever and persistent Q fever. It identified five hub genes (IL4, IL6, IL1B, CD28, and AKT1) that may contribute to the progression of Q fever by regulating immune responses. These findings provide new insights into the immunopathogenesis of Q fever and offer potential directions for its diagnosis and treatment.

## Supporting information

S1 File LM22 signature matrix. This file contains the reference gene expression profiles of 22 human immune cell subsets, which serves as the core input reference for the CIBERSORT algorithm in immune infiltration analysis. The matrix provides specific gene expression signatures for each immune cell type, enabling the deconvolution of mixed cell populations to quantify the relative proportions of the 22 immune cell subsets via support vector regression. S2. List of immune-related genes (IRGs) with a GeneCards score > 10. These genes were identified by querying the term “immune” in the GeneCards database (https://www.genecards.org/).
(TXT)
